# Role of Treatment Theory and Enablement Theory for Restoring Health and Rehabilitation in Saudi Arabia

**DOI:** 10.7759/cureus.9180

**Published:** 2020-07-14

**Authors:** Abdulaziz M Alsufyani

**Affiliations:** 1 Nursing, Ministry of Human Resources and Social Development, Taif, SAU

**Keywords:** enablement theory, treatment theory, theoretical framework

## Abstract

Several challenges are involved in providing the appropriate treatment during the process of a patient’s rehabilitation. Studies conducted in relation to the rehabilitation issues are ineffective in providing useful developments in the provided treatments. Most of the studies lack the utilization of theoretical framework which ultimately proposed weak findings. This study aimed to provide a theoretical framework in relation to the treatment theory as well as the enablement theory along with their effectiveness in improving rehabilitation treatments. The study involves a thorough review undertaking of published articles from 2014 to 2019 that were based on the rehabilitation and other health restoration measures through PubMed and Medline databases. Findings of the study were proposed in the form of a theoretical framework of treatment and the enablement theory. For rehabilitation and clinical researches, it is recommended to use treatment theory and enablement theory to propose unique results that may contribute to the improved healthcare treatment.

## Introduction and background

The clinical practices regarding a patient’s rehabilitation and health restoration involve various challenges and highly complex clinical interventions. The understanding regarding the problems and challenges in clinical practices is important and can only be catered through the development of theoretical framework along with its successful implementation in the practical situations [1]. The ideas are highly relatable in clinical settings, as they contribute to the development of scientific knowledge regarding patient’s health and rehabilitation. Rehabilitation practices are common in almost every region. The increased ratio of people aging with significant disabilities attracts the interest of researches in discussing the usefulness of rehabilitation practices and their impact in restoring patient’s health by the intervention of highly successful practices [2].

In most of the developing countries, there lies a significant gap between the practical implementation of theories, as rehabilitation profession, poor educational level, and lack of resources to give more contest are still underrated and are not regulated successfully. Other important dimensions include the effectiveness of the patient’s social, psychological, cultural, and environmental factors that are highly ignored during the assessment of the patient’s health condition leading towards the decision-making process [3]. The improvement of the healthcare quality is highly related to the above factors along with other factors associated with the medical condition of the patients [1]. In Saudi Arabia the healthcare services are provided following the hierarchy of public which is classified into three levels, i.e. primary, secondary, and tertiary. Primary care is associated with healthcare preventive measures, rehabilitation, and treatment services including healthcare and environmental education. In secondary care, an organized approach has been adopted concerning the activities and responsibilities of every healthcare department. The final level, i.e. tertiary healthcare services is associated with the diseases that are at an advanced stage, where treatment measures are undertaken following the state-of-the-art approach [4].

In the case of rehabilitation treatments, theory-guided researches are sometimes challenging as they contribute to different domains that are important with respect to the rehabilitation. To provide the effective results, understanding regarding the effectiveness and the differences between the two theories is important. The utilization of treatment theories is important, as they provide important knowledge in relation to the alternate treatment methods.

The fundamental target of the rehabilitation treatments is associated with the clinical phenomenon that ranges from attaining the basic knowledge regarding the patient’s body structure, by using advance treatment devices to the responsibility of arranging the psychoeducational programs for patients belonging to any age or disability [5]. Daley associated the idea with the evolution of two highly related theories that have been developed to support the healthcare field [6]. The theories include: treatment theory and the enablement theory [7]. The two theories are important, as they make an important contribution to the research and practices undertaken in rehabilitation centers.

Keith and Lipsey were the first to define treatment theory, according to which it refers to the nature of the treatment process that is effective in transforming the treatment therapy into health conditions [8]. Dijkers et al. elaborated the idea referring treatment theory to the class of theories that are used to specify clinical mechanism by which the active constituents of the provided treatment are altered [9]. As a result of this, the target functions of the body are achieved. Hart et al. provided further developments in treatment theory, according to which it is divided into tripartite structure [10]. The first includes treatment ingredients which refer to the chosen treatment interventions by the clinical expert. Second is associated with the mechanism through which the action is being undertaken. It deals with the expectations of the healthcare experts with the provided treatment. The final part refers to the target, which focuses on the treatment target in improving the patient’s health.

Next includes the enablement theory that is associated with the effects of treatments undertaken and their remote but highly essential aspects. It is associated with significant predictions regarding how the patient’s impairment or disability may affect the treatment activities. The central focus of the theory is associated with the idea as to how patient’s restrictions or limitations might be changed. It is highly associated with the understanding regarding different factors that are important in influencing patient’s treatment success [11]. A well-developed enablement theory is potentially strong to guide and predict the performance of different complex skills of patients [12].

Problem statement

It has been more than a decade that researchers along with the multidisciplinary group of clinicians are working to develop effective solutions that may improve the treatment measures for patient’s health restoration and rehabilitation [10]. Several studies have been conducted regarding the effectiveness of rehabilitation treatments and services provided to patients [9, 12-13]. However, little knowledge is available regarding the theoretical framework of treatment theory and enablement theory along with their contributions in restoring health and rehabilitation. To provide the important developments in treatment measures, the analysis regarding the effectiveness of two theories is important.

Study aim

The study aims to provide a theoretical framework in relation to the treatment theory and enablement theory and their effectiveness in improving healthcare and rehabilitation treatments in Saudi Arabia. Contributions of this study are important in improving the healthcare treatments by infusing significant developments.

Methods

An in-depth search regarding the structured literature was undertaken through PubMed and Medline by using different terminologies like, ‘rehabilitation’, ‘theoretical framework’, ‘health restoration’, ‘treatment theory’, and ‘enablement theory’. The abstracts and keywords of the studies, proposed from January 2014 to 2019 were studied. The selection of the studies was based on the inclusion criteria, according to which studies that are specifically associated with rehabilitation and health restoration in Saudi Arabia or in Gulf Cooperation Council served as the part of discussion. Whereas, studies conducted in any other region were excluded. Due to limited studies, only 15 were identified with the added keywords in search. Finally, five studies were reviewed and included in the study as they were fully complied based on the inclusion criteria.

## Review

Results and discussion

Effectiveness of Treatment Theory and Enablement Theory

The motivation for a novel treatment might make use of both enablement and treatment theories; that is, one may identify that the function of most patients is restricted by several collaborative factors, but might concentrate on the progression of an enhanced or new treatment for merely one of these factors. Understanding the two theories is important to provide important recommendations for an effective treatment. Treatment theory is important as it contributes to the structural taxonomy of the provided treatments in rehabilitation centers. The effectiveness of the treatment theory lies in the fact that it focuses on the individual understanding and realization for the value of the provided treatment. This type of understanding is formed by focusing on different factors that cease the potential ability of the treatment.

Yousef conducted a study to determine different barriers that are responsible for the social inclusion of disabled population in Saudi Arabia [14]. The factors were based on different dimensions including the social, cultural, financial, and socioeconomic conditions. The fundamental focus of the examination was associated with the probabilities of making addition to the existing policies that are favorable in the rehabilitation of the patients with disabilities. The study used quantitative methods for data collection, where participants including disabled people were interviewed. Findings were provided following different themes including the quality of life, problems and perceptions, Saudi law, policies and their implementation, etc. The major focus of this study was granted to Social Work Theory, Labelling Theory, and the Humanistic theory [15-17]. The study illustrated several limitations. Among them, one of the major limitations was related to the lack of theoretical interventions that might support different knowledge associated with the rehabilitation of physically disabled people. The study suggested the need to conduct a quantitative study to identify the extent of the problems. It further suggested the need of providing the disability statistics followed by the mixed method research. Despite the significant findings the study lacks the use of two highly useful theories, i.e. enablement theory and treatment theory that are important in the context of health management and rehabilitation theory that may add value to the proposed results. With the adoption of these theories, Yousef can highlight the issues in several areas such as nonpayment of financial support, lack of high-quality medical care, provisions and services, issues with mobility, negative attitudes and perceptions from society, and medical negligence [14].

Bindawas and Vennu provided a detailed insight regarding the stroke rehabilitation; as in Saudi Arabia, the problem contributes as one of the major health issues leading towards severe disability or even death [18-20]. The overall study is based on the theoretical knowledge regarding different causes, effects, and treatment interventions regarding the given problem. Findings of the study indicated that Saudi Arabia lacks the necessary measures in providing quality treatment to the affected patients. The study provided some important suggestions for future researchers, according to which the rehabilitation must address the rate of prevalence in different geographical regions of Saudi Arabia, role of environment in the development of stroke, and affirmation regarding the use of ICF model in Saudi Arabia. One of the major lackings of this study is its inability to discuss the topic following the intervention of treatment theory which is valuable in developing some useful conclusions that are valuable for clinical professionals as well as for those suffering from stroke.

Meny and Hayat assessed clinical professionals’ knowledge of occupational therapy in Makkah [21]. A quantitative design was followed to recruit 320 healthcare professionals including: nurses, physicians, social workers, and physical therapists working in different hospitals of Saudi Arabia. Findings of the study indicated that most of the healthcare professionals have limited or poor knowledge regarding the use and effectiveness of occupational therapy [22]. Findings further indicated that among the four groups, the highest knowledge was attained by physicians, then physical therapists, and nurses. The only limitation involved in this study is that data were collected from the tertiary care government hospitals.

In addition, Alshomrani conducted a cross-sectional study to investigate the implementation of important elements of therapeutic communities (TC) in Saudi TC between September 2014 and December 2014 [23]. Survey of Essential Elements Questionnaire (SEEQ) was conducted for data collection. Findings of the study indicated significantly low scoring in the six dimensions of SEEQ, including; TC perspective, approach and structure of agency treatment, community working as the therapeutic agent, work and educational activities, process and formal therapeutic elements. The study suggested that low scoring in the given dimensions indicated the need of conducting further studies that are important in identifying the causes and solutions of the problems. Further, it highlights the need to conduct a longitudinal study depending on outcome-based evidences. Despite the detailed findings, the study could not provide evidences in support of the theoretical interventions that might be helpful in providing the extended knowledge in the given framework.

Furthermore, AlJohi et al. conducted a study to identify different barriers in the successful cancer rehabilitation of Saudi patients [24]. The study was conducted at King Fahad Medical City, where data were collected through semi-structured focus groups and interviews from participants including allied healthcare professionals and physicians. Content analysis was performed, leading towards the development of key themes that include: the knowledge and competency of healthcare providers, communication barriers, limited rehabilitation services available for cancer patients along with other barriers that are associated with patients and their families. The findings of the study suggested that the major barriers in the given context are related to the ineffectiveness of clinical practice patterns that restricts the success of cancer rehabilitation treatments. The study involves few limitations as the factors discussed by the healthcare professionals were in general context. The study was restricted in terms of involving healthcare clinics found within the region. The aim of the study was useful; however, the identification of factors in support of the theoretical interventions may help in providing more concrete justifications in relation to different barriers associated with rehabilitation treatment. It would be further helpful in making a useful addition to the current policies and the rehabilitation guidelines to increase the treatment efficacy.

However, the role of treatment theory and the enablement theory is of greater importance as it contributes to improving the treatment modalities in relation to the specific characteristics of patients. As stated above an effective study contributes to proposing visible advantages in terms of the treatment effectiveness. The management of any disease depends upon the treatment provided and includes routine work in the form of treatment. These tasks are undertaken to detect any chances of the recurrence of the treatment along with other symptoms that are effective in progression of the treatment. Treatment theory provides a meaningful way of determining the treatment and the mode of treatment, as it targets which ingredient of the provided treatment may be used to improve the treatment. Enablement theory is introduced in the later stage of the treatment to increase the generalizability of the study.

Theoretical Framework and Analysis

Treatment theory and enablement theory are important when contributing to the literary studies related to rehabilitation and healthcare treatments. The intervention of the two theories is important to improve the treatment conditions for rehabilitation in Gulf Cooperation Council (GCC) countries. Most of the studies discussed the idea considering other related theories, and are still unsuccessful in determining the valid treatment measures that are effective in providing the ultimate benefit in terms of restoring human health.

Enablement theory appears massively when guiding research on eventual treatment utility once efficacy at the level of the treatment target is explained; that is, the question changes from “Does the treatment have the presumptious impacts on the target?” to “In what types of patients does use of this treatment have significant functional advantages?” The investigators potentially to innovate in the realm of exercise treatment are those with adequate knowledge of metabolism and muscle physiology, and of the differential effects of different types of muscular challenge. On the contrary, there is minimal evidence to consider these investigators would be well-informed regarding the role of balance, and how the strength demands differ with patients' pre-requisites. Thereby, a handoff from those investigators is required for comprehensive maturation of rehabilitation treatments from those investigators to focus on treatment theory as compared to enablement theory. However, contributions of treatment theory as well as the enablement theory are important in providing important recommendations for instance; X treatment is useful for patients with X characteristics.

A treatment taxonomy, in this regard helps in providing useful guidelines for the treatment research. Both treatment theory and the enablement theory are productive in proposing the valuable results with respect to the provided theory and the expectations regarding the developments in the provided treatment. Most of the studies such as Bindawas and Vennu, Meny and Hayat, Alshomrani, and AlJohi et al. discussed different issues in relation to various health restoration problems that are highly dependent upon the usefulness of the provided treatment [18, 21, 23-24]. This further indicates that the present health system still lacks the ability to provide proper treatment measures for rehabilitation treatments. Implication of treatment as well as the enablement theory is important in this regard as it would help to control the growing ratio of health-related problems. Treatment theory specifically helps in predicting the outcomes of the given treatment in terms of the patient’s response. It is further helpful in providing useful instances regarding the usefulness of changing the direct object of the treatment [25].

Most of the studies are conducted following an aim to identify the treatment efficacy provided to a patient with certain disease. Studies that are majorly dependent on the treatment concerns of the patient’s health recovery must be conducted by employing the treatment and enablement theory.

As for instance, Yousef conducted a study to determine different barriers that are responsible for the social inclusion of the disabled population [14]. Findings of the study were based on the labeling theory, humanistic theory, and social work theory. Major focus in this study was provided to the involvement of the changes in policies that are effective in removing the barriers.

Gipson and King provided a study determining the value of theories in relation to health-related behaviors when providing treatment to the suicidal individuals [26]. A significant focus was granted to the theory of planned behavior along with the intervention of the Health Belief model. The study further discussed about the problems that are encountered during the provision of the treatment to highly depressed individuals who are more towards the suicidal attempts. Role of treatment and enablement theories is of significant value here, as they are effective in providing useful propositions that are effective in providing useful methods that will be in accordance to the psychology of such patients. As indicated by Whyte, the absence of the treatment theory makes clinical studies to focus on a single treatment, as treatment study targets the changes in treatment ingredients that might alter the mechanism of the resulting actions [27]. Treatment theory further enables to develop a science of the treatment efficiency. As every treatment provided is limited to only a small group of individuals based on their characteristics, the intervention of the treatment theory enables the researcher to make useful hypothetical assumptions about which treatment is best suited for which sort of individuals, leading towards the proposition of useful findings through hypothesis testing.

The usefulness of the treatment and the enablement theories is not only limited to a certain instance, as indicated by Whyte and Barrett [7]. The treatment theory is highly critical about the rigorous designs that are involved in clinical research. This specifically takes place during the initial phase of the treatment development [28-29]. Treatment theory is also useful in terms of developing a valid methodology in guiding the researcher for the development of the inclusion and the exclusion criteria. The formulation of the treatment and action mechanism enables the researcher to identify the types of patients that are more responsive towards the provided treatment. Another advantage of the treatment theory is that it helps in selecting the most valid outcome measures, as the initial critical outcome helps in determining the change in the target treatment [28]. It is further effective in developing a useful design of the explanatory trials that are of greater importance during the decision-making process.

Treatment theory is also effective in specifying the treatment active ingredients of the provided treatment and thus offers basis for developing the foundation for the evaluation of the treatment adherence, so as to analyze the degree to which the subjects receive the active ingredients. Similarly, enablement theory contributes to the progression of the study and serves as the useful component of the rehabilitation researches. It specifically contributes to the later phases of the formation of valid treatment [30]. It further contributes to providing the generalizability criteria for the provided treatment along with its benefits [12].

As for instance, Bindawas and Vennu conducted a study to provide a detailed insight regarding stroke rehabilitation [18]. Overall developments of the study are based on the causes and the efficiency of different treatment interventions provided to patients. The intervention of the treatment and enablement theories would be effective in proposing the useful findings in relation to the treatments that are specific to certain population. In addition, it would enable the researcher to indicate the generalizability of the proposed findings as stated above.

Most of the clinical and rehabilitation researches that aim to provide a useful contribution to the existing literature lack important developments in terms of providing useful categorization of treatments with their findings. Treatment theory in this regard is effective in providing findings that have a strong association with the clinical decision-making process [31]. In most of the studies associated with clinical and rehabilitation researches, the final propositions are based on the intervention of the enablement theory, as it determines the effectiveness of the treatment over distal clinical outcomes. Treatment and the enablement theories together help the researcher to propose useful findings in terms of the benefits of the provided treatment. Whyte added that using treatment theory helps the researcher to propose clinical outcomes that are effective in satisfying the practical needs of the rehabilitation process [28]. Any treatment remains ineffective unless it proposes some useful findings that are effective in improving the treatment activity, increases participation while improving the quality of life of patients. These demands can only be satisfied when highly effective findings are proposed.

Consider the study of AlJohi et al. , who examined different barriers in the successful cancer rehabilitation [24]. The study used a qualitative approach, and results are proposed in themes. Such result remains ineffective until they serve an ultimate benefit to the treatment interventions. Fishbein emphasized the value of theory-based intervention programs as they are effective in reducing the risks of unhealthy treatment outcomes, while maximizing the chances of developing healthy behaviors among the affected individuals [32]. The results of clinical studies that are supported by the treatment and enablement theories help in providing useful guidelines in relation to treatment researches. Alshomrani, investigated the implementation of the important elements of the TC that affect the efficacy of the treatment [23]. Findings indicated structure and approach of the agency treatment as one of the important factors that may affect the effectiveness of the TC. Intervention of the treatment theory and the enablement theory is important in this case to propose more concrete and filtered results in terms of the provided treatment and the response of patients over the provided treatment.

However, theories are effective in proposing useful suggestions on the part of the clinical experts who are effective in controlling the development and high prevalence of disease [33]. Also, the theories discussed here are not new to the clinical discourse, but are always relevant in the Saudi healthcare context due to timely changes. Clinical trainees are usually asked by their supervisors for articulating the empirical evidence that a treatment is influential or providing a presumptious rationale for the forecasted influence of a treatment. Clinical trainees are also usually challenged with questions including, “Well, this treatment may work, but will it have any functional effect?” These types of questions are not usually clearly articulated even though such questions typically correspond to enablement theory and treatment theory. Acknowledging the difference between enablement and treatment theory assists clinicians in identifying those treatments that have empirical support in order to alter their preferred target, and those patient attributes that recognize the clinical benefits from those treatments such as guided by the enablement theory. Clinical education will be consequently improved through continued research on each domain and their interrelation.

These recommendations are helpful in minimizing the time spent on a single patient, as clinical experts, through most of the clinical rehabilitation studies might develop the idea regarding the most suitable treatment for the patient. The implication of the two theories is important in achieving the aim of the rehabilitation studies, while providing the usefulness of the treatment. Contributions of both theories are thus important in the rehabilitation treatment researches to provide the new improved developments in the given area of study.

## Conclusions

Theory-guided research is specifically contrasting in rehabilitation due to the wide range of domains that are examined by rehabilitation and the subsequently diverse set of concepts that is appropriate. However, the role of treatment theory and the enablement theory is of greater importance as it contributes to improving the treatment modalities in relation to the specific characteristics of patients. These different types of theory overcome fundamentally different challenges on how to persuade change and how to forecast the distal effects of that change. Clinical education, practice, and research can be mutually advanced by understanding their separate contributions and learning for navigating them.
